# Endovascular treatment of uterine myomatosis: a systematic review

**DOI:** 10.1590/1677-5449.190149

**Published:** 2020-07-06

**Authors:** Kamilla Rosales Costa, Patrick Bastos Metzger

**Affiliations:** 1 Escola Bahiana de Medicina e Saúde Pública – EBMSP, Salvador, BA, Brasil.; 2 Hospital Universitário Professor Edgar Santos – HUPES, Salvador, BA, Brasil.; 3 Hospital Cárdio Pulmonar – HCP, Salvador, BA, Brasil.

**Keywords:** myoma, leiomyoma, fibroma, uterine artery embolization

## Abstract

Uterine Artery Embolization (UAE) is a noninvasive alternative to open surgery for treatment of uterine myomatosis. This study aims to analyze the efficacy and safety of UAE in these cases. A systematic review was carried out of studies available on the Medline (via PubMed) and the LILACS and PEDro (via the Biblioteca Virtual em Saúde) databases. The searches found 817 studies, 7 of which were selected according to the eligibility criteria (analytical, longitudinal, retrospective, or prospective studies), with a total of 367 patients studied. The variables analyzed and the characteristics of the studies included were collated and input to a database. Rates of volume reduction of the uterus and the dominant myoma were 44.1% and 56.3%, respectively. Mean rate of complete infarction of the dominant myoma was 88.6% (82-100%). The mean number of complications observed was 15±8.6 cases, most of which were classified as minor, and no deaths were recorded. The mean number of re-interventions in absolute values was 12.2±15.5 cases. Therefore, in the literature analyzed, uterine artery embolization is an effective procedure with a low rate of complications for treatment of uterine leiomyomatosis.

## INTRODUCTION

Uterine leiomyomatosis is the most common cause of morbidity in women of fertile age.^1^^,^^2^ Its incidence varies widely, depending on ethnicity and age, with rates varying from 5 to 80%. It can be treated clinically or surgically and this choice should consider size and location. Conventional surgical treatments, hysterectomy and myomectomy, are the most frequently performed interventions because of their efficacy with relation to both the tumor and its symptomatology.^3^^,^^4^ Uterine artery embolization (UAE) has recently emerged as a less invasive option for treatment of uterine myomatosis. The technique consists of injection of polymer microspheres or polyvinyl-alcohol particles into both uterine arteries by catheterization via the femoral artery or the radial artery. The procedure thus causes selective ischemia of the myomatous tissue by cessation of arterial flow, without injuring the uterine parenchyma.^1^^,^^5^

Comparative studies of UAE against the standard treatment have reported controversial results for the efficacy of the procedure. Advantages of embolization described in the literature include shorter duration of surgery and faster recovery after the procedure, with consequent earlier return to activities, shorter length of hospital stay, and lower frequency of immediate complications caused by the minimally invasive technique, in addition to lower morbidity compared with other techniques.^1^^,^^5^^-^7 The rate of complications varies, the most common of which are expulsion of the myoma and ovarian dysfunctions with consequent changes to follicle stimulating hormone (FSH).^1^^,^^6^

The objective of this study is to analyze the efficacy of UAE in terms of reduction of the volume of the uterus and of the dominant myoma, in addition to its safety, in terms of rates of complications and re-interventions.

## METHODS

This is a systematic review of the literature, conducted in accordance with the Preferred Reporting Items for Systematic Reviews and Meta-Analyses (PRISMA) methodology.^8^ The article is based on secondary data and does not require submission to the Research Ethics Committee for approval.

Searches were run on the electronic databases MEDLINE (via PubMed), Literatura Latino-Americana e do Caribe em Ciências da Saúde – LILACS, and Physiotherapy Evidence Database – PEDro (via Biblioteca Virtual em Saúde – BVS). Articles published from 2009 to 2014 were identified using a combination of keywords from the Descritores em Ciências da Saude (DeCS^i^) and Medical Subject Headings (MeSH^ii^) platforms. Studies in which patients with uterine myomatosis, whether symptomatic or not, were treated using UAE were selected for the review.

**Figure 1:**
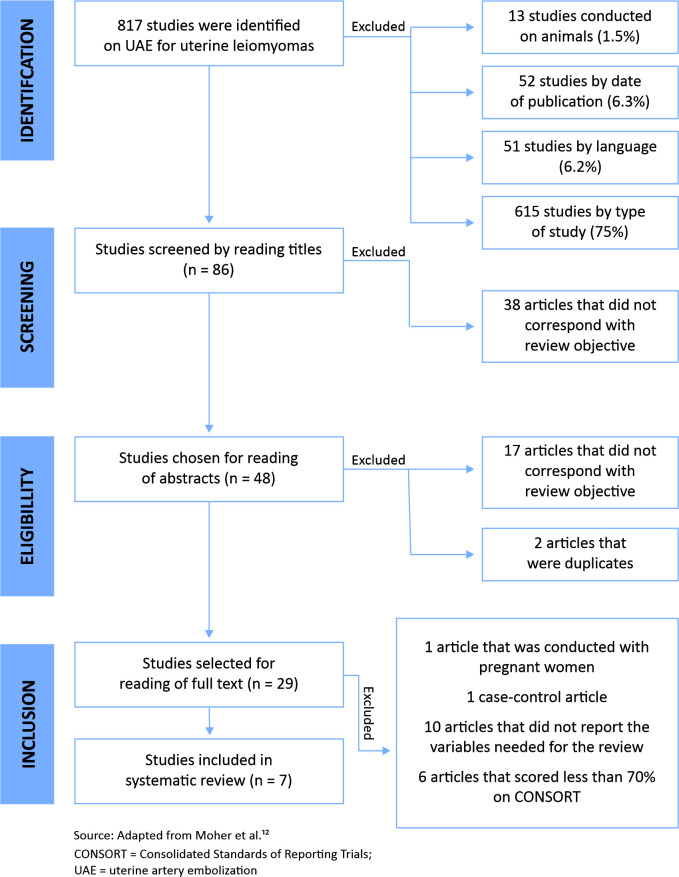
Flow diagram illustrating identification, screening, eligibility, and inclusion of studies in the systematic review. n = number of patients in the sample.

All studies identified on the databases were included if they investigated women over the age of 18 years and were published during the last 10 years in Portuguese, English, or Spanish, and used a clinical trial or cohort study design. Studies were excluded if they were case reports, guidelines, duplicates, systematic reviews, or letters to the editor, did not assess endovascular treatment for uterine myomatosis, or were conducted with pregnant women (Figure 1).

Studies were graded for methodological quality using assessment of risks of bias as set out in the Strengthening the Reporting of Observational Studies in Epidemiology (STROBE)^9^ guidelines, for cohort studies, or in the Consolidated Standards of Reporting Trials (CONSORT)^10^ guidelines, for clinical trials. Articles were considered of acceptable quality if they met at least 70% of the criteria in the respective evaluation tool.

After analysis and selection of articles, data were collected from those that had not been excluded, extracting the following variables: title, authors, year of publication, country, sample size, mean age of patients, initial volume of the dominant myoma, reduction in volume of the uterus and/or of the dominant myoma after the procedure, myoma infarction rate, complications after the procedure, and need for re-interventions. Variables were input to and stored in Excel spreadsheets.

## RESULTS

A total of 817 studies were identified. After screening and application of the eligibility criteria and grading for methodological quality, a total of seven articles compatible with the study objective and of satisfactory quality were selected, making a combined total sample of 367 patients (Figure 1). All of these studies were analytical, longitudinal, retrospective, or prospective, and published from 2009 to 2014 in English (Table 1).^11^^-^17

**Table 1:** General characteristics of the studies analyzed.

**Authors**	**Year and country of publication**	**Study design**	**Objective**	**Sample size**	**Embolization sample**
Hald et al.^11^	2009, Norway	Randomized clinical trial	To compare long-term recurrence of symptoms and magnetic resonance results at 6 months after two different treatments for leiomyomas.	n = 58	n = 26
Mara et al.^12^	2012, Czech Republic	Non-randomized clinical trial	To compare the results of two different types of occlusive treatment for uterine myomas.	n = 200	n = 100
Shlansky-Goldberg et al.^13^	2014, United States	Randomized clinical trial	To assess the efficacy of two embolic agents for treatment of symptomatic uterine leiomyomas.	n = 60	n = 60
Smeets et al.^14^	2010, Netherlands	Cohort	To retrospectively analyze the long-term results of uterine artery embolization in symptomatic women with a large myoma burden.	n = 71	n = 71
Sone et al.^15^	2010, Japan	Non-randomized clinical trial	To assess the safety and efficacy of uterine artery embolization with gelatin sponge for symptomatic leiomyomas.	n = 33	n = 33
Song et al.^16^	2013, South Korea	Non-randomized clinical trial	To compare clinical and magnetic resonance results after uterine artery embolization with non-spherical polyvinyl alcohol versus gelatin sponge particles.	n = 60	n = 60
Vilos et al.^17^	2014, United Kingdom	Cohort	To assess efficacy and post-procedure pain associated with uterine artery embolization using Gelfoam alone versus Embospheres plus Gelfoam in women with symptomatic uterine myomas.	n = 17	n = 17

n = number of patients in the sample.

Mean initial volume of the dominant myoma was 244 cm^3^. The efficacy of uterine artery embolization was assessed in terms of three variables: reduction in volume of the uterus (44.1+5.9%), reduction in volume of the dominant myoma (56.3±7%), and rate of complete infarction of the dominant myoma (88.6±6.9%) **(** Table 2).^11^^-^17

**Table 2:** Data related to efficacy of uterine artery embolization in the studies reviewed.

**Study**	**Initial volume of the dominant myoma (cm^3^)**	**Reduction in volume of the uterus**	**Reduction in volume of the dominant myoma**	**Complete infarction of the dominant myoma**
Hald et al.^11^	257 (35-530)	51.3±15.4% (after 6 months)	62.8±27% (after 6 months)	100% (after 6 months)
Mara et al.^12^	188.7±39.6/14-630	NR	53% (after 6 months)	82% (after 6 months)
Shlansky-Goldberg et al.^13^	203.3±275.1 (PVA)	NR	NR	82.1% (PVA)
	141.1±179.6 (TAG)			85.7% (TAG)
				(after 3 months)
Smeets et al.^14^	450 (42-1265)	43%	44%	86%
Sone et al.^15^	321 (64-1922)	NR	61.4% (after 12 months)	NR
Song et al.^16^	184.1±141.3 (nPVA)	46.8±11.4% (after 3 months)	60.2±18.1% (after 3 months)	96±7% (3 months)
	265.3±339 (Gelform)			
Vilos et al.^17^	144.3 (44-299)	35.3% (after 12 months)	NR	NR
	(Gelform) 286 (41-603)			
	(Gelform + Embospheres)			
Mean	244 cm^3^	44.1±5.9%	56.3±7%	88.6±6.9%

NR = not reported; TAG = *Tris*-Acryl*gelatina; nPVA =* Nonspherical polyvinyl alcohol; PVA = Polyvinyl alcohol microspheres.

All of the studies that reported complications provided these data in absolute values, with a total of 75 events (23.5%) (Table 3).^11^^-^17 The mean number was 15±8.64 cases, the majority of which were classified as minor complications, and there were no deaths recorded. The most common complications in the studies were amenorrhea (transitory or permanent) and expulsion of the myoma (Table 4).^11^^-^17

**Table 3:** Distribution of patients by presentation of perioperative and postoperative complications

	Hald et al.^11^	Mara et al.^12^	Shlansky-Goldberg et al.^13^	Smeets et al.^14^	Sone et al.^15^	Song et al.^16^	Vilos et al.^17^
Number of patients with complications	NR	28 (28%)	3 (5%)	21 (29.5%)	12 (36.4%)	11 (18.3%)	NR
Mean	23.5%						

NR = not reported.

**Table 4:** Frequency of common perioperative and postoperative complications in studies.

**Types of complications**	Hald et al.^11^	Mara et al.^12^	Shlansky-Goldberg et al.^13^	Smeets et al.^14^	Sone et al.^15^	Song et al.^16^	Vilos et al.^17^
Transitory amenorrhea	NR	-	-	5 (7%)	6 (18.2%)	1 (1.7%)	NR
Permanent amenorrhea	NR	-	-	5 (7%)	1 (3%)	-	NR
Expulsion of the tumor	NR	7 (7%)	1 (1.7%)	1 (1.4%)	1 (3%)	3 (5%)	NR

NR = not reported.

The choice of type of procedure employed in reinterventions for uterine myomatosis was made on the basis of the patients’ profiles, their preferences, and the hospitals’ protocols, and the predominant choices made were to repeat UAE or use the already well-established techniques of hysterectomy and myomectomy. One hysteroscopic endometrial ablation was performed, but was unrelated to the burden of myomas (Table 5).^11^^-^17 The mean rate of reinterventions, in absolute values, was 12.2±15.5 cases.

**Table 5:** Distribution of patients by surgical re-interventions.

**Study**	**Hysterectomy**	**UAE**	**Myomectomy**	**Hysteroscopic endometrial ablation**
Hald et al.^11^	2 (8%)	-	-	-
Mara et al.^12^	-	1 (1%)	36 (36%)	-
Shlansky-Goldberg et al.^13^	1 (1.7%)	-	-	-
Smeets et al.^14^	10 (14%)	8 (11.3%)	-	-
Sone et al.^15^	NR	NR	NR	NR
Song et al.^16^	NR	NR	NR	NR
Vilos et al.^17^	2 (12%)	-	-	1 (6%)

UAE = uterine artery embolization; NR = not reported.

## DISCUSSION

Uterine artery embolization is a minimally invasive procedure used to treat benign tumors of the uterus as an alternative to conventional therapy for symptomatic women who wish to preserve their fertility, menstrual flow, and uterus.^1^ The procedure’s advantages include treatment of a larger number of myomas in a single intervention, earlier return to daily activities and employment activities, and reduced incidence of complications and need for blood transfusions.^1^^,^^18^^,^^19^

This systematic review was conducted to analyze the efficacy of UAE for treatment of uterine leiomyomatosis and describe the incidence rates of postoperative complications and re-interventions. Two cohort studies and five clinical trials with a total sample of 367 patients were analyzed.

Pron et al.^20^ reported a greater reduction in volume of the uterus after embolization and a larger baseline uterus volume, which was not observed in this systematic review. In our study, we found that Vilos et al.^17^ reported the smallest reduction in uterine volume out of all of the studies included (35.3%). However, median uterine volumes were 144.3 cm^3^ (Gelfoam embolization) and 286cm^3^ (embolization with Gelfoam + embospheres). A study by Shlansky-Goldberg et al.^13^ reported a mean uterine volume reduction 3 months after embolization of 436.4 cm^3^±352.1 cm^3^ for a group treated with polyvinyl alcohol (PVA) microspheres and 557.8 cm^3^±1101.1 cm^3^ for a group treated with tris-acryl gelatin microspheres (TAG). The study also reported mean reductions in volume of the dominant myoma 3 months after embolization: 76.9 cm^3^±135.8 cm^3^ for the PVA group and 27.4 cm^3^±42.3 cm^3^ for the TAG group. Hald et al.^11^ exhibited the greatest reduction in uterine volume (51.3%), but they reported a median of 257 cm^3^. This finding may be because of differences in the follow-up periods in these studies, since Hald et al.^11^ followed patients for 6 months, whereas Vilos et al.^17^ followed theirs for 12 months. Additionally, the methodology used by Vilos et al.^17^ did not involve randomization, which could affect patient selection and, consequently, the reduction in uterine volume after the procedure.

The reduction in uterine volume reported by the studies included ranged from 35.3% to 51.3%. The mean reduction in uterine volume for all studies was 44.1%±5.9. These findings are compatible with the conclusions of Katsumori et al.,^21^ who observed reductions in uterine volume in the range of 35 to 60%, depending on the degree of infarction of the dominant myoma. In a later study by Katsumori et al.,^22^ reductions in uterine volume of 49.8 to 54.3% were reported after 12 months’ follow-up of patients, which is the same period as in Vilos et al.^17^ Nevertheless, Vilos et al.^17^ reported a smaller reduction in uterine volume (35.3%). These studies have different types of design: a prospective cohort study and a non-randomized clinical trial, respectively. Furthermore, Katsumori et al.^22^ studied a considerably larger sample (n = 152) than Vilos et al.^17^ (n = 17), which also had a smaller initial volume of the dominant myoma. These differences during the study may have influenced the findings on the efficacy of the procedure.

Another way of analyzing the efficacy of the method is to monitor the change in volume of the dominant myoma, since this measure provides information directly related to the influence of the procedure on the tumor, excluding changes to healthy uterine tissues. Reductions in volume of the dominant myoma reported in the literature range from 41 to 68%^21^^,^^23^ and are compatible with the results of this systematic review, in which the mean reduction was 56.3%±7.

The factors that lead to myoma infarction are not fully understood. Notwithstanding, it is known that morphology, level of collateral blood supply, and technical details (embolic agent, embolization outcome, and operator experience) can be directly linked with the degree of infarction of these tumors.^24^ Several studies have investigated differences in efficacy of UAE conducted using different embolic agents and even using different diameter particles; but they did not detect statistically relevant differences in effectiveness.^13^^,^^16^^,^^17^^,^^24^

Rates of complete infarction of the dominant myoma vary considerably in the literature, from 35 to 91.7%.^20^^,^^24^ In the present review, the mean rate observed was 88.6%±6.9, but higher rates of complete infarction than previously reported were observed. Hald et al.^11^ and Song et al.^16^ achieved 100% and 96%, respectively. In both studies, the procedure was performed by experienced operators, using 355-500 µm PVA particles. Hald et al.^11^ also reported that the majority of the myomas treated with UAE were classified as intramural. In contrast, the study by Song et al.^16^ did not record this information.

Complications related to endovascular treatment of uterine myomatosis may be the results of changes provoked at the puncture site in the femoral or iliac artery; of arterial injuries; or of obstructions caused by the guidewire, catheters, or clots, or even by inadvertent embolizations of other blood vessels.^1^ Complications can be classified as minor, when they do not require hospital admission or special care, or major, when hospitalization is necessary and complications could cause the patient’s death.

The most common complications were expulsion of the myoma and amenorrhea. The latter is described as possibly related to patient age and could be limited to a few menstrual cycles (transitory) or not. This effect on the menstrual cycle, associated with ovarian failure, is caused by unintended migration of embolic particles into the ovarian circulation, which reduces its blood flow, with consequent hypoestrogenism and endometrial atrophy, and can culminate in premature menopause (amenorrhea persistent).^1^^,^^18^ The literature describes transitory amenorrhea rates of around 10%. In turn, depending on the age group of patients, permanent amenorrhea rates can reach 3% among women up to the age of 45 years, or as high as 15% among older patients.^1^^,^^25^

Transitory amenorrhea was reported in three of the studies reviewed, Smeets et al.,^14^ Sone et al.,^15^ and Song et al.,^16^ with relatively low rates and a delay before return of menstruation of around 3 months. In contrast, Smeets et al.^14^ reported permanent amenorrhea in five patients (one patient aged less than 43 years and another four aged over 47 years), and Sone et al.^15^ reported permanent amenorrhea in one patient, whose age was not stated, but in whom FSH levels were monitored and exhibited increase at 12 months.

Ovarian failure and consequent cessation of menstrual flow can also be caused by technical failures during the procedure, such as inadequate embolization of the uterine-ovarian anastomoses, by anatomic variants, such as ovaries predominantly fed by the uterine arteries, or even by exposure to ionizing radiation.^1^^,^^26^

Expulsion of the myoma is another possible complication associated with UAE, which, in some cases, requires surgical removal to resolve the condition.^18^^,^^26^ Faria et al.^26^ recorded a 10% rate of myoma expulsion among embolized patients. This complication was reported by the authors of all of the studies included in the present review.

The mean number of reinterventions in absolute values was 12.2±15.5 cases. Analyzing the absolute numbers, it can be observed that Mara et al.^12^ and Smeets et al.^14^ reported the highest numbers of surgical re-interventions, at 37 and 18, respectively, and also the lowest rates of reduction in volume of the dominant myoma, at 53 and 44%, in that order. The lower the reduction of dominant myoma volume, the higher the risk of re-intervention.^27^^-^29

This study has three limitations. First, there were a small number of articles available with good methodological quality that were compatible with the subject investigated. It is also a limitation that these articles had different length follow-up periods, reducing the possibilities for comparison of values between them. Finally, there is the issue of different imaging methods for diagnosis and monitoring of uterine myomatosis, transvaginal ultrasonography or pelvic magnetic resonance, which have different levels of accuracy.

## CONCLUSIONS

Uterine artery embolization offers effective treatment for women with uterine myomatosis who wish to preserve their uterus or who are at high risk from conventional surgery. Complications related to the procedure are classified as minor and of low incidence. Re-interventions are relatively frequent after endovascular treatment and are intimately related to the course of the underlying disease.

## Footnotes

i DeCS: (Mioma OR Leiomioma) AND (Embolização da artéria uterina).

ii MeSH: ("Myoma" OR "Leiomyoma" OR "Fibroma") AND ("Uterine Artery Embolization").
