# Supplementary material for: Endovascular treatment of uterine myomatosis: a systematic review
Source: J Vasc Bras. 2020 Jul 6;19:e20190149. doi: 10.1590/1677-5449.190149 (PMC8202181; doi:10.1590/1677-5449.190149)
Supplement: Translation

# Translation: Tratamento endovascular da miomatose uterina: uma revisão sistemática

**Authors:** Kamilla Rosales Costa, Patrick Bastos Metzger

**Affiliations:** 1 Escola Bahiana de Medicina e Saúde Pública – EBMSP, Salvador, BA, Brasil.; 2 Hospital Universitário Professor Edgar Santos – HUPES, Salvador, BA, Brasil.; 3 Hospital Cárdio Pulmonar – HCP, Salvador, BA, Brasil.

**Keywords:** mioma, leiomioma, embolização da artéria uterina

## Abstract

A embolização da artéria uterina (EAU) é um tratamento pouco invasivo e alternativo à cirurgia aberta no tratamento de miomatose uterina. Este estudo visa analisar a eficácia e a segurança da EAU nesses casos. Para isso, foi realizada uma revisão sistemática a partir de estudos disponíveis nas bases de dados MEDLINE/PubMed, LILACS e PEDro, via Biblioteca Virtual em Saúde. Foram encontrados 817 trabalhos; destes, 7 foram selecionados pelos critérios de elegibilidade (estudos analíticos, de corte longitudinal, retrospectivos ou prospectivos), totalizando 367 pacientes no estudo. As variáveis estudadas, bem como as características dos estudos incluídos, foram coletadas e armazenadas em um banco de dados. As taxas de redução do volume uterino e do mioma dominante foram 44,1% e 56,3%, respectivamente. A média do infarto completo do mioma dominante foi de 88,6% (82-100%). Quanto às complicações, a média obtida foi de 15±8,6 casos, sendo a maioria destas classificadas como menores, e nenhum óbito foi registrado. A média de reabordagem em valores absolutos foi de 12,2±15,5 casos. Portanto, a embolização da artéria uterina é um procedimento eficaz e com baixa taxa de complicações para o tratamento da leiomiomatose uterina na literatura analisada.

## INTRODUÇÃO

A leiomiomatose uterina constitui a causa mais comum de morbidade em mulheres em idade fértil^1^^,^^2^. Tem incidência muito variável de acordo com etnia e idade, com números variando entre 5 e 80%. O tratamento pode ser clínico ou cirúrgico, e essa escolha deve considerar o tamanho e a localização. O tratamento cirúrgico convencional, histerectomia e miomectomia, é a intervenção mais realizada devido à eficácia com relação ao tumor e à sintomatologia^3^^,^^4^. A embolização da artéria uterina (EAU) surgiu recentemente como opção de tratamento menos invasivo da miomatose uterina. A técnica consiste na introdução de microesferas de polímero ou partículas de polivinil-álcool em ambas as artérias uterinas através da cateterização da artéria femoral ou da artéria radial. Com isso, o procedimento gera uma isquemia seletiva no tecido miomatoso por meio da cessação do fluxo arterial, sem causar lesão do parênquima uterino^1^^,^^5^.

Estudos comparativos entre EAU e o tratamento padrão revelaram resultados controversos quanto à eficácia do procedimento. Entre as vantagens da embolização, são relatados na literatura menor tempo operatório e de recuperação após o procedimento, com consequente retorno mais rápido às atividades, menor tempo de internação e menor frequência de complicações imediatas decorrentes da técnica minimamente invasiva, além da menor morbidade comparada às demais técnicas^1^^,^^5^^-^7. A taxa de complicações varia, sendo as mais comuns parturição do mioma e disfunções ovarianas com consequente alteração dos níveis de hormônio folículo-estimulante (FSH)^1^^,^^6^.

Este estudo objetiva analisar a eficácia da EAU quanto à redução do volume uterino e do mioma dominante, bem como sua segurança, relativa às taxas de complicações e de reabordagens deste procedimento.

## MÉTODOS

Trata-se de uma revisão sistemática da literatura conduzida de acordo com a metodologia Preferred Reporting Items for Systematic Reviews and Meta-Analyses (PRISMA)^8^. Este trabalho utiliza dados secundários, não necessitando de submissão e aprovação do Comitê de Ética em Pesquisa para o desenvolvimento.

As buscas foram feitas nas bases de dados eletrônicos MEDLINE (via PubMed), Literatura Latino-Americana e do Caribe em Ciências da Saúde – LILACS e Physiotherapy Evidence Database – PEDro (via Biblioteca Virtual em Saúde – BVS). Foram pesquisados artigos publicados no período de 2009 a 2014, por meio da combinação de descritores obtidos nas plataformas Descritores em Ciências da Saúde (DeCS^i^) e Medical Subject Headings (MeSH^ii^). Os estudos selecionados foram aqueles em que pacientes portadoras de miomatose uterina, sintomática ou não, foram submetidas à EAU como tratamento.

Foram incluídos todos os trabalhos encontrados nas plataformas de dados realizados em mulheres, com idade superior a 18 anos, publicados nos últimos 10 anos, em português, inglês ou espanhol, cujos desenhos de estudo sejam ensaios clínicos ou de coorte. Foram excluídos os estudos de relato de caso, *guidelines*, duplicados, revisões sistemáticas, cartas a editor, que não avaliaram o tratamento endovascular da miomatose uterina ou que foram realizados em gestantes (Figura 1).

**Figura 1:** Fluxograma de identificação, triagem, elegibilidade e inclusão de estudos na revisão sistemática. n = número de pacientes na amostra.

Os estudos foram avaliados quanto à qualidade metodológica de acordo com a avaliação do risco de viés, através da utilização do Strengthening the Reporting of Observational Studies in Epidemiology (STROBE)^9^ para os estudos de coorte e do Consolidated Standards of Reporting Trials (CONSORT)^10^ para os ensaios clínicos. Foram considerados artigos de qualidade aceitável aqueles que preencheram ao menos 70% dos critérios da ferramenta de avaliação.

Após análise e seleção dos artigos, a coleta de dados dos que restaram foi iniciada, extraindo as seguintes variáveis: título, autores, ano de publicação, país, número da amostra, média de idade das pacientes, volume inicial do mioma dominante, redução do volume uterino e/ou do mioma dominante após o procedimento, taxa de infarto do mioma, complicações após o procedimento e necessidade de reabordagens. As variáveis foram coletadas e armazenadas em planilhas do Excel.

## RESULTADOS

Foram identificados 817 trabalhos. Após a triagem e a aplicação dos critérios de elegibilidade, bem como a avaliação da qualidade metodológica, foram selecionados sete artigos compatíveis com o objetivo do estudo e de qualidade satisfatória, totalizando uma amostra de 367 pacientes (Figura 1). Todos esses estudos foram do tipo analítico, de corte longitudinal, retrospectivos ou prospectivos, publicados entre os anos de 2009 e 2014 e em língua inglesa (Tabela 1 11^-^17).

**Tabela 1:** Características gerais dos estudos analisados.

**Autores**	**Ano e país de publicação**	**Desenho do estudo**	**Objetivo**	**Tamanho da amostra**	**Amostra da embolização**
Hald et al.^11^	2009, Noruega	Ensaio clínico randomizado	Comparar a recorrência dos sintomas a longo prazo e resultados de ressonância magnética (RM) de 6 meses após dois tratamentos diferentes para os leiomiomas uterinos.	n = 58	n = 26
Mara et al.^12^	2012, República Checa	Ensaio clínico não randomizado	Comparar os resultados de dois tipos diferentes de terapia oclusiva de miomas uterinos.	n = 200	n = 100
Shlansky-Goldberg et al.^13^	2014, Estados Unidos	Ensaio clínico randomizado	Avaliar a eficácia de dois agentes embólicos no tratamento de leiomiomas uterinos sintomáticos.	n = 60	n = 60
Smeets et al.^14^	2010, Países Baixos	Coorte	Avaliar retrospectivamente os resultados a longo prazo da embolização da artéria uterina (EAU) em mulheres sintomáticas com uma grande carga de miomas.	n = 71	n = 71
Sone et al.^15^	2010, Japão	Ensaio clínico não randomizado	Avaliar a segurança e a eficácia da EAU com esponja de gelatina para leiomiomas sintomáticos.	n = 33	n = 33
Song et al.^16^	2013, Coreia do Sul	Ensaio clínico não randomizado	Comparar os resultados clínicos e de ressonância magnética (MRI) após EAU com álcool polivinílico não esférico (nPVA) *versus* partículas de esponja de gelatina.	n = 60	n = 60
Vilos et al.^17^	2014, Londres	Coorte	Avaliar a eficácia e a dor pós-procedimento associada à EAU usando Gelfoam sozinho *versus* Embospheres mais Gelfoam em mulheres com miomas uterinos sintomáticos.	n = 17	n = 17

n = número de pacientes na amostra.

O volume inicial médio dos miomas dominantes foi de 244 cm^3^. A eficácia da embolização da artéria uterina foi avaliada através de três variáveis: redução do volume uterino (44,1+5,9%), redução do volume do mioma dominante (56,3±7%) e taxa de infarto completo do mioma dominante (88,6±6,9%) **(** Tabela 2 11^-^17).

**Tabela 2:** Dados relacionados à eficácia da embolização da artéria uterina nos diferentes estudos.

**Estudo**	**Volume inicial do mioma dominante (cm^3^)**	**Redução do volume uterino**	**Redução do volume do mioma dominante**	**Infarto completo do mioma dominante**
Hald et al.^11^	257 (35-530)	51,3±15,4% (6 meses após)	62,8±27% (6 meses após)	100% (6 meses após)
Mara et al.^12^	188,7±39,6/14-630	NR	53% (6 meses após)	82% (6 meses após)
Shlansky-Goldberg et al.^13^	203,3±275,1 (PVA)	NR	NR	82,1% (PVA)
	141,1±179,6 (TAG)			85,7% (TAG) (3 meses após)
Smeets et al.^14^	450 (42-1265)	43%	44%	86%
Sone et al.^15^	321 (64-1922)	NR	61,4% (12 meses após)	NR
Song et al.^16^	184,1±141,3 (nPVA)	46,8±11,4% (3 meses após)	60,2±18,1% (3 meses após)	96±7% (3 meses)
	265,3±339 (Gelform)			
Vilos et al.^17^	144,3 (44-299)	35,3% (12 meses após)	NR	NR
	(Gelform) 286 (41-603)			
	(Gelform + Embospheres)			
Média	244 cm^3^	44,1±5,9%	56,3±7%	88,6±6,9%

NR = não referido; TAG = Microesferas de gelatina tris; nPVA = Álcool polivinílico não esférico; PVA = Microesferas de álcool polivinílico.

Todos os estudos que relataram complicações forneceram esses dados em valores absolutos, com um total de 75 eventos (23,5%) (Tabela 3 11^-^17). A média obtida foi de 15±8,64 casos, sendo a maioria classificada como complicações menores, e nenhum óbito foi registrado. As mais comuns entre os estudos foram a amenorreia (transitória ou permanente) e expulsão/parturição do mioma (Tabela 4 11^-^17).

**Tabela 3:** Distribuição de pacientes quanto à apresentação de complicações perioperatórias e pós-operatórias.

	Hald et al.^11^	Mara et al.^12^	Shlansky-Goldberg et al.^13^	Smeets et al.^14^	Sone et al.^15^	Song et al.^16^	Vilos et al.^17^
Número de pacientes com complicações	NR	28 (28%)	3 (5%)	21 29,5%)	12 (36,4%)	11 (18,3%)	NR
Média	23,5%						

NR = não referido.

**Tabela 4:** Frequência de complicações perioperatórias e pós-operatórias comuns aos estudos.

**Tipos de complicações**	Hald et al.^11^	Mara et al.^12^	Shlansky-Goldberg et al.^13^	Smeets et al.^14^	Sone et al.^15^	Song et al.^16^	Vilos et al.^17^
Amenorreia transitória	NR	-	-	5 (7%)	6 (18,2%)	1 (1,7%)	NR
Amenorreia permanente	NR	-	-	5 (7%)	1 (3%)	-	NR
Expulsão do tumor	NR	7 (7%)	1 (1,7%)	1 (1,4%)	1 (3%)	3 (5%)	NR

NR = não referido.

A escolha do tipo de procedimento realizado para reabordagem da miomatose uterina foi feita de acordo com o perfil das pacientes, suas preferências e o protocolo dos hospitais, havendo uma predominância de escolha pela repetição da EAU e pelas técnicas já bem consolidadas (histerectomia e miomectomia). Uma ablação endometrial histeroscópica foi realizada, mas não estava relacionada à carga de miomas (Tabela 5 11^-^17). A média de reabordagem em valores absolutos foi de 12,2±15,5 casos.

**Tabela 5:** Distribuição de pacientes quanto a reabordagens cirúrgicas.

**Estudo**	**Histerectomia**	**EAU**	**Miomectomia**	**Ablação endometrial histeroscópica**
Hald et al.^11^	2 (8%)	-	-	-
Mara et al.^12^	-	1 (1%)	36 (36%)	-
Shlansky-Goldberg et al.^13^	1 (1,7%)	-	-	-
Smeets et al.^14^	10 (14%)	8 (11,3%)	-	-
Sone et al.^15^	NR	NR	NR	NR
Song et al.^16^	NR	NR	NR	NR
Vilos et al.^17^	2 (12%)	-	-	1 (6%)

EAU = embolização da artéria uterina; NR = não referido.

## DISCUSSÃO

A EAU é um procedimento minimamente invasivo utilizado para o tratamento de tumores benignos do útero, sendo uma alternativa à terapia convencional para mulheres sintomáticas que desejam preservar a fertilidade, o fluxo menstrual e o útero^1^. Entre as vantagens deste procedimento, estão o tratamento de um maior número de miomas em uma única abordagem, a reintrodução às atividades cotidianas e profissionais mais precoce e a redução da incidência de complicações, bem como da necessidade de hemotransfusões^1^^,^^18^^,^^19^.

Esta revisão sistemática buscou analisar a eficácia da EAU para o tratamento da leiomiomatose uterina, bem como descrever a incidência de complicações e reabordagens pós-operatórias. Foram incluídos dois estudos de coorte e cinco ensaios clínicos, totalizando 367 pacientes na amostra.

Pron et al.^20^ relacionaram, em seu trabalho, uma maior redução do volume uterino pós-embolização a um maior volume uterino de base das pacientes, relação esta que não foi observada na revisão sistemática. Em nosso estudo, verificamos que Vilos et al.^17^ apresentaram a menor redução do volume uterino entre os estudos contemplados (35,3%). No entanto, apresentaram uma mediana de volume uterino de 144,3 cm^3^ (Gelfoam) e 286 cm^3^ (Gelfoam + embosferas). O estudo de Shlansky-Goldberg et al.^13^ apresentou a média de redução do volume uterino 3 meses após a embolização: 436,4 cm^3^ ± 352,1 cm^3^ para o grupo de microesferas de álcool polivinílico (PVA), 557,8 cm^3^ ± 1101,1 cm^3^ para o grupo de microesferas de gelatina tris (TAG). Informou ainda a média de redução do volume do mioma dominante 3 meses após a embolização: 76,9 cm^3^ ± 135,8 cm^3^ para o grupo PVA, 27,4 cm^3^ ± 42,3 cm^3^ para o grupo TAG. Hald et al.^11^ apresentaram a maior redução do volume uterino (51,3%); entretanto, relataram uma mediana de 257 cm^3^. Este achado pode ser atribuído à diferença de tempo de seguimento entre os estudos, visto que Hald et al.^11^ acompanharam suas pacientes por 6 meses, enquanto Vilos et al.^17^ acompanharam por 12 meses. Além disso, este último estudo não apresentou randomização em sua metodologia, podendo influenciar na seleção dos pacientes e, consequentemente, na redução do volume uterino após o procedimento.

A redução do volume uterino relatada nos estudos incluídos variou de 35,3% a 51,3%. A média de redução do volume uterino dentre os estudos foi de 44,1%±5,9. Estes achados são compatíveis com as conclusões de Katsumori et al.^21^, que encontraram reduções do volume uterino variando entre 35-60% dependendo do grau de infarto do mioma dominante. Em trabalho posterior de Katsumori et al.^22^, foi demonstrada uma redução do volume uterino entre 49,8-54,3% ao final de 12 meses de acompanhamento das pacientes, período compatível com o de Vilos et al.^17^. No entanto, os achados de mudança no volume uterino deste último mostram-se inferiores (35,3%). Esses estudos apresentaram desenhos diferentes: coorte prospectiva e ensaio clínico não randomizado, respectivamente. Além disso, Katsumori et al.^22^ apresentaram uma amostra consideravelmente maior (n = 152) quando comparada à amostra de Vilos et al.^17^ (n = 17), que apresentou ainda um volume inicial do mioma dominante inferior. Essas diferenças durante a execução do estudo podem influenciar os achados da eficácia do procedimento.

Outra forma de analisar a eficácia do método é o acompanhamento da alteração do volume do mioma dominante, já que essa medida informa diretamente a influência do procedimento sobre o tumor, excluindo a alteração dos tecidos uterinos saudáveis. A redução do volume do mioma dominante é relatada na literatura em torno de 41 a 68%^21^^,^^23^, dados compatíveis com os resultados dessa revisão sistemática, cuja média foi de 56,3%±7.

O infarto do mioma não tem seus fatores de desenvolvimento bem compreendidos. No entanto, acredita-se que a sua morfologia, o grau de suprimento sanguíneo colateral e os detalhes técnicos (agente embólico, desfecho da embolização e experiência do operador) possam estar diretamente ligados ao grau de infarto desses tumores^24^. Diversos trabalhos avaliaram as diferenças na eficácia da EAU com diferentes agentes embólicos e até mesmo diferentes diâmetros de partículas; entretanto, não encontraram divergências estatisticamente relevantes quanto à efetividade^13^^,^^16^^,^^17^^,^^24^.

As taxas de infarto completo do mioma dominante variam consideravelmente na literatura, de 35 a 91,7%^20^^,^^24^. Nesta revisão, pôde-se observar uma média de 88,6%±6,9; identificaram-se, porém, taxas de infarto completo superiores às que já foram relatadas anteriormente. Hald et al.^11^ e Song et al.^16^ alcançaram 100% e 96%, respectivamente. Ambos os estudos tiveram o procedimento conduzido por operadores experientes e utilizaram partículas de PVA de 355-500 µm. Hald et al.^11^ descreveram ainda que a maior parte dos miomas abordados por EAU foram classificados como intramurais. O estudo de Song et al.^16^, no entanto, não registrou tal informação.

As complicações relacionadas ao tratamento endovascular da miomatose uterina podem ser provenientes de alterações no sítio de punção da artéria femoral ou ilíaca, lesões arteriais, obstruções causadas pelo fio-guia, cateteres ou coágulos ou ainda por embolizações indevidas de outros vasos sanguíneos^1^. As complicações podem ser classificadas em menores, quando não necessitam de internamentos ou cuidados especiais, ou maiores, quando o internamento é necessário e as complicações podem levar a paciente ao óbito.

As complicações mais comuns foram expulsão do mioma e amenorreia. Esta última é descrita como possivelmente relacionada à idade da paciente, podendo se limitar a alguns ciclos menstruais (transitória) ou não. Essa alteração do ciclo menstrual associada à falência ovariana é justificada pela migração indevida de partículas embólicas para a circulação ovariana, que leva à diminuição da sua irrigação com consequente hipoestrogenismo e atrofia endometrial, podendo culminar em menopausa precoce (amenorreia persistente)^1^^,^^18^. A literatura descreve uma taxa de amenorreia transitória em torno de 10%. Já a amenorreia permanente varia de acordo com a faixa etária das pacientes, podendo ser de 3% entre as mulheres de até 45 anos ou de até 15% nas pacientes com idade superior^1^^,^^25^.

A amenorreia transitória foi relatada por três trabalhos – Smeets et al.^14^, Sone et al.^15^ e Song et al.^16^ –, com taxas consideravelmente baixas e período de reversão em torno de três meses. Por outro lado, Smeets et al.^14^ relataram amenorreia permanente em cinco pacientes (uma paciente com idade inferior a 43 anos e outras quatro acima de 47 anos), e Sone et al.^15^, em uma paciente cuja idade não foi informada, mas na qual os níveis de FSH foram medidos e demonstraram elevação aos 12 meses.

A falência ovariana com consequente cessação do fluxo menstrual pode ser causada ainda por falhas técnicas do procedimento, como embolização inadequada das anastomoses útero-ovarianas, por variações anatômicas – ovário de irrigação predominantemente realizada pelas artérias uterinas ou ainda por exposição à radiação ionizante^1^^,^^26^.

A expulsão ou parturição do mioma é outra possível complicação associada à EAU, necessitando, em alguns casos, de remoção cirúrgica para resolução do quadro^18^^,^^26^. Faria et al.^26^ registraram em seu estudo uma taxa de expulsão de mioma de 10% entre as pacientes embolizadas. Nesta revisão, pôde-se observar que essa complicação foi relatada por todos os autores.

A média de reabordagem em valores absolutos foi de 12,2±15,5 casos. Ao analisar os números absolutos, pôde-se observar que Mara et al.^12^ e Smeets et al.^14^ apresentaram os maiores valores de reabordagens cirúrgicas – 37 e 18, respectivamente – e as menores taxas de redução do volume do mioma dominante – 53 e 44%, nesta ordem. Quanto menor a redução do volume do mioma dominante, maior o risco de reabordagens^27^^-^29.

As limitações deste trabalho são três. Primeiro, há um pequeno número de artigos disponíveis com boa qualidade metodológica e compatíveis com o tema proposto. Também consiste como uma limitação o fato de que os artigos têm diferentes durações de períodos de acompanhamento das pacientes, diminuindo a possibilidade de comparação dos valores entre eles. Por último, a questão dos diferentes métodos de imagem para diagnóstico e acompanhamento da miomatose uterina – ultrassonografia transvaginal e ressonância magnética pélvica, que apresentam diferentes valores de acurácia, também é uma limitação.

## CONCLUSÃO

A embolização da artéria uterina possibilita um tratamento eficaz para mulheres com miomatose uterina, que tenham o interesse de preservar o útero ou que apresentem riscos elevados para a realização das cirurgias convencionais. As complicações do procedimento são classificadas como menores e de baixa incidência. As reabordagens são relativamente frequentes após o tratamento endovascular e estão intimamente relacionadas com a evolução da doença de base.

## Footnotes

i DeCS: (Mioma OR Leiomioma) AND (Embolização da artéria uterina).

ii MeSH: ("Myoma" OR "Leiomyoma" OR "Fibroma") AND ("Uterine Artery Embolization").
